# Study on the physical and chemical properties of lead passivating agent in soil

**DOI:** 10.1038/s41598-023-45567-5

**Published:** 2023-10-25

**Authors:** Chengyu Han, Juan Li, Jianglong Shen

**Affiliations:** 1 School of Human Settlements and Civil Engineering, Xi’an Jiaotong University, Xi’an, 710049 China; 2 Shaanxi Engineering Research Center of Land Consolidation, Xi’an, 710075 China

**Keywords:** Environmental sciences, Solid Earth sciences, Physical chemistry

## Abstract

With the rapid development of industry, heavy metal pollution has seriously damaged the health of soil, and heavy metals spread through the food chain, posing a threat to human health. The firm existence of heavy metals in soil under earthy conditions is a center trouble faced by soil dense metal pollution solidification and correction technology. However, the existing investigation results are mostly controlled to soil passivation experiments using various materials. Macroscopically, heavy metal passivation materials have been selected, but the intrinsic mechanisms of different compound functional groups in soil passivation have been ignored. With the common heavy metal ion Pb^2+^ as an example, the stability of the combination of heavy metal ions and common ion groups in soil was analyzed in this study by using quantum chemical calculation as the theoretical guidance. The results show that SO_4_^2−^ and PO_4_^3−^, as functional groups of passivating agents, are used to control lead pollution and have been verified to have good effects. When the pollution is particularly serious and not easy to passivation and precipitation, Fe^3+^ can be considered to enhance the passivation effect.

## Introduction

Lead (Pb) has been widely reported in globally polluted soils, and Pb pollutants are one of the top ten types of pollutants that pose the greatest threat to human health, and are considered one of the primary potential toxic elements (PTES)^1,2^. Excessive lead in soil can cause many toxic symptoms to plants. This includes delayed growth and development, chlorosis, root blackening, inhibition of photosynthesis, mineral nutrient disorders, water imbalance, hormone changes, and effects on membrane structure and permeability^3,4^. Soil plays a fundamental utility in food sureness. The adverse effects of contaminants, such as heavy metal(loid)s, on crop attribute have portended human health. Lead is not degraded and excreted in the human body, but accumulates in various body organs and blood. As the lead content exceeds the acceptable standards of the human body, it can harm human health to a certain extent, leading to a series of serious illnesses, especially irreversible toxicity to children's physiological and intellectual development^5,6^. Consequently, it is essential to pay high attention to the fact that lead will not be degraded and excreted in the human body, But it accumulates in various body organs and blood of the human body. As the lead content exceeds the acceptable standard of the human body, it will to some extent endanger human health, leading to a series of serious illnesses^7,8^. The remediation technologies of heavy metal pollution in soil are mainly divided into physical remediation, chemical remediation and bioremediation^9,10^. The common physical remediation methods mainly include soil replacement, but they are only applicable to the remediation of small areas of contaminated soil. Common bioremediation technologies mainly include phytoremediation, animal remediation, and microbial remediation; Although this method has the advantages of economy and efficiency, phytoremediation takes a long time and cannot repair deeply polluted soil. Animal restoration has a very limited scope due to the limited range of animal activity^11^. Microbial remediation is seriously affected by the environment and microbial diversity. Chemical remediation refers to the addition of chemicals to contaminated soil to react with heavy metals, thereby achieving the goal of reducing the toxicity of heavy metals^9^. Lead, as the most biologically toxic and active heavy metal in soil, has been widely studied for its insitu passivation technology in polluted soil, and passivation materials are becoming increasingly abundant. At present, commonly used materials mainly include calcium silicate materials, lime materials, phosphate mixtures, animal manure, sepiolite, zeolite, bentonite and various soil amendments^12^. The conceptual optimal solution for the rectification of heavy metal pollution from agricultural nonpoint source is adding amendments to contaminated soil to change the heavy metal elements from the bioavailable state to the residual state, limit the migration of heavy metal ions, and realize in-situ rectification. For the research and improvement of amendments, understanding the physical and chemical attribute of heavy metal pollutants is of great value, and the key research content is to understand the solidification, adsorption, and chemical action of such polluted heavy metals under natural temperature and pressure.

At present, the nature of soil lead pollution is mainly determined through experiments. Previous studies have confirmed that phosphate and sulfur containing substances can be used as stabilizers for lead^13^. Although it is possible to experimentally determine the beneficial effects of some materials on soil passivation, it remains to be studied which substances in the materials have an impact and how they do so. The different natural states of soil affect the precipitation conditions of lead containing compounds. Therefore, the stability problem of lead containing compounds cannot be fundamentally solved using traditional chemical theories. This paper attempts to evaluate the stability of common ions binding to Pb^2+^ by using quantum geochemical tools.

At present, theoretical research on the energy stability of different lead compounds is rarely reported. First-principles computing has been widely used in numerous scientific research directions, for instance the study of unknown material properties in materials science^14,15^. For example, calculating the photoelectric properties of substances could guide the design and synthesis of photoelectric materials with strong functions^16,17^. In geoscience, first-principles are used to imitate structure and composition^18^. In thermodynamics, the simulation fabrication and configuration of minerals were studied^19^. The research on passivating agents used for insitu remediation of heavy metal pollution is mainly focused on testing the passivation of contaminated soil using different substances or doses of passivating agents.

This paper aimed to calculate and solve the theoretical existence state of the heavy-metal element lead in soil by means of quantum geochemical tools and soil environmental analysis data. On the basis of the calculation results, the physical and chemical conditions that interfere with the existence state of lead and the modified stable existence state were further deduced. Common acid ions, such as chloride ions, carbonate ions, silicate ions, and sulfate ions, and common metal cations, such as aluminum ions and iron ions, were introduced into the calculation of lead-containing system by applying first- principles. In this manner, the stability of the combination of functional groups and Pb^2+^ could be further understood to design more suitable passivating agents for soil repair later. The existing soil lead pollution control is mostly concentrated in the direction of quantitative testing. By studying the crystal structure of lead compounds and experimentally analyzing the complexation reaction between lead-contaminated soil and different ions, this paper aims to explore the internal mechanism of lead-contaminated soil passivation and provide a more accurate direction for chemical treatment of soil heavy metal pollution.

## Materials and methods

Density functional theory (DFT) was proposed by Hohenberg and Kohn in 1964. DFT, as a quantum mechanical method to study multi electron systems, its main idea is to use the electron density of the system to describe the physical and chemical properties of the system. Nowadays, density functional theory, as an important means, has been widely used to describe the ground state problems of multi particle systems. This paper used the first principle of density functional theory to calculate. The calculation software was Venna Ab initio Simulation Package (VASP). Kohn Sham exchange correlation can use GGA-PBE^20,21^. In this paper, the kinetic energy cut-off energy of plane wave vector basis set is 500 eV.

In this paper, the crystal coefficient file or the corresponding crystal was first required in the crystal library, processed by the CATSEP plate in Materials Studio, and then placed into the VASP package for first-principles calculation^22–24^. Using projection plus wave method for projection, PAW_ The pseudopotential of PBE as a projection plus wave is the energy function of PBE exchange key. The electron orbitals of Pb were corrected by the + U treatment of the 3d electron orbitals and calculated accordingly. The parameter settings in the calculation were as follows: precession was set as accurate, and stopping-criterion for electronic upd = 1.E−04 conversion unit was set to eV, that is, the convergence point energy limit is 10^−4^, corresponding to the convergence energy limit between atoms is 10^−2^ eV/nm. In the optimization process, the optimized NSW = 999 was selected, that is, the number of steps in the nucleus was 999, and the subsequent self-consistent motion in the nucleus remained immobile in the nucleus, that is, NSW = 0. The calculation of each system was carried out after the convergence test, in which the parameters of the truncated energy and the Brillouin zone position point (K point) were set in accordance with the different calculation systems, and the Monkhorst–Pack method was adopted for the selection of K points^25–27^. The plane wave truncation energy was first optimized for the value of the equal difference series 200, 250, 300, 350, 400, 450, 500, 550, and 600 eV because the structural optimization part and the energy calculation part have different requirements for accuracy.

The dilation width of Pb in the evaluation parameter setting was selected as SIGMA = 0.05. In the process of K point selection, considering the precision and evaluation cost, the optimization process was as large as possible when the calculation accuracy allows, and the value of K point was relatively reduced when calculating the state density. A extremely symmetric K-point spatial arrangement was used when calculating energy bands.

## Results

### Crystal structure selection and optimization

**Figure 1:**
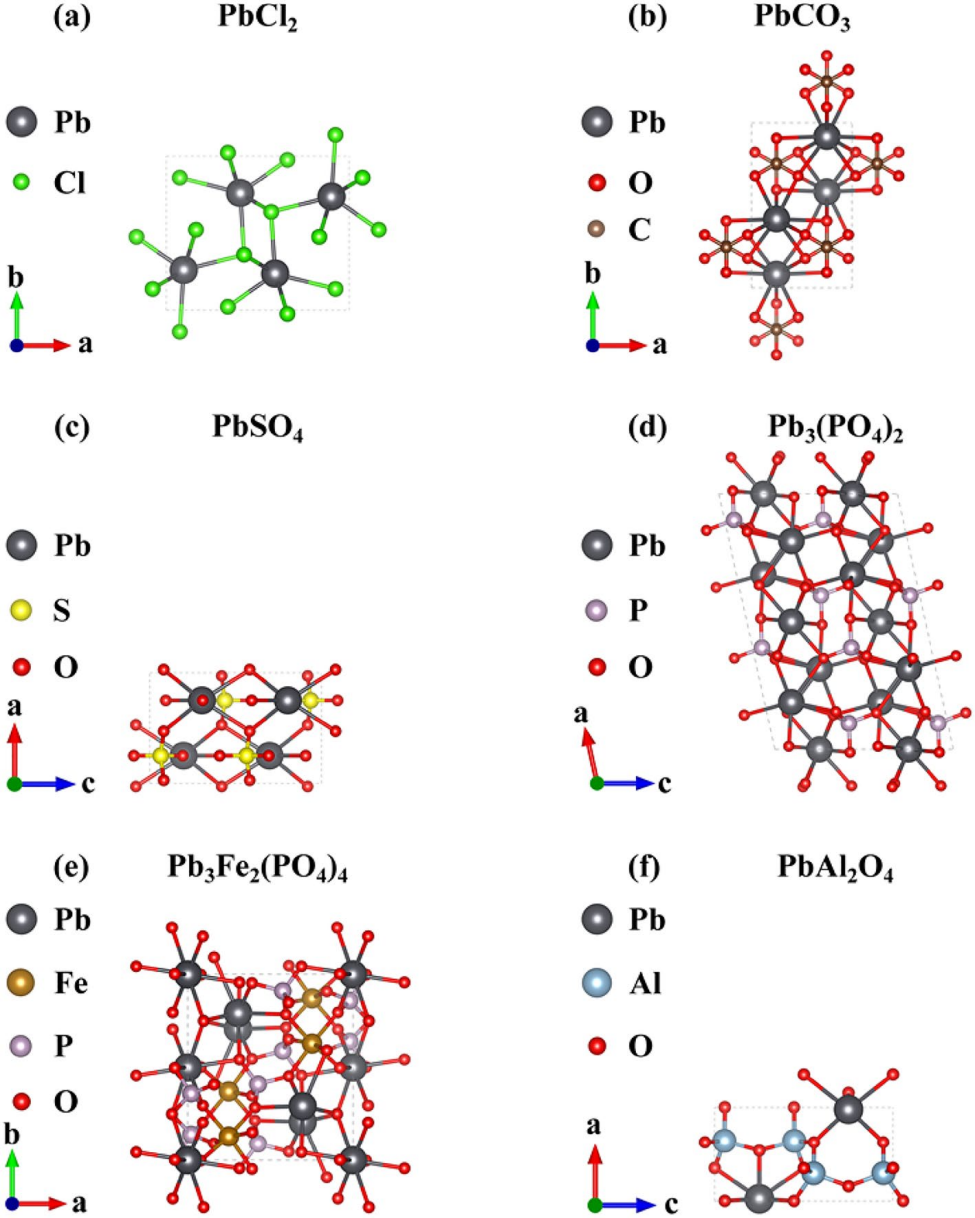
Unit cell structure of PbCl_2_, PbCO_3_, PbSO_4_, Pb_3_(PO_4_)_2_, Pb_3_Fe_2_(PO_4_)_4_, and PbAl_2_O_4._

After the difficulty of obtaining Pb compound groups, the purity level, and the process level was comprehensively considered, the types of Pb-containing crystals used for calculation were selected to provide reference for the selection of passivators in the later stage. The ion group was selected from chloride ion, carbonate, sulfate, phosphate, iron ion, and aluminum ion, and the corresponding chemical formulas were PbCl_2_ (Lead chloride), PbCO_3_(Lead carbonate), PbSO_4_(Lead(II) sulfate), Pb_3_(PO_4_)_2_(Lead phosphate), Pb_3_Fe_2_(PO_4_)_4_, and PbAl_2_O_4_, respectively. The crystal structure corresponding to the above compounds was obtained from the Crystallography Open Database (Fig. 1). First, the stability of each anionic group binding to lead was discussed on the basis of the lead compounds PbCl_2_, PbCO_3_, PbSO_4_, and Pb_3_(PO_4_)_2_ bound by four anion groups, and the address of various ions in the soil was further combined. Al^3+^ and Fe^3+^ were introduced to form complex lead compounds to explore whether the addition of common metal cations could enhance the stability of the system.

Table 1 shows the unit cell structure parameters obtained after optimization of four Pb compounds. These unit cell parameter values optimized by VASP calculation were very close to the ideal experimental value, and the change ratio was within 0.2%, which further indicated that the first-principle calculation of VASP is the same as the experimental result under ideal conditions. The calculated working parameters used in this paper are reasonable and credible.

**Table 1:** Unit cell parameters for PbCl_2_, PbCO_3_,PbSO_4_,Pb_3_(PO_4_)_2_,Pb_3_Fe_2_(PO_4_)_4_,PbAl_2_O_4._

Model	Lattice constant (Å)			Shaft angle			Volume (Å^3^)
	a	b	c	α	β	γ	
PbCl_2_	9.03	7.608	4.525	90	90	120	310.8686
PbCO_3_	5.1834	8.4992	6.1475	90	90	120	270.8165
PbSO_4_	5.5218	7.0993	8.5608	90	90	90	335.595
Pb_3_(PO_4_)_2_	13.799	5.6917	9.4196	90	90	90	722.6685
Pb_3_Fe_2_(PO_4_)_4_	9.1349	9.1349	9.468	90	118	90	708.6814
PbAl_2_O_4_	5.0782	5.2432	8.4361	90	90	90	196.57

### Free energy

The energy system of PbCl_2_, PbCO_3_, PbSO_4_, Pb_3_(PO_4_)_2_, Pb_3_Fe_2_(PO_4_)_4_, and PbAl_2_O_4_ at room temperature and pressure was calculated by VASP (Table 2). The Gibbs free energy of the six lead-containing systems was sorted from high to bottom as PbCl_2_, PbAl_2_O_4_, PbCO_3_, PbSO_4_, Pb_3_(PO_4_)_2_, and Pb_3_Fe_2_(PO_4_)_4_. The relative stability of these systems was inversely correlated with their corresponding Gibbs free energy ordering. The relative structural stability order of the lead compounds was Pb_3_Fe_2_(PO_4_)_4_, Pb_3_(PO_4_)_2_, PbSO_4_, PbCO_3_, PbAl_2_O_4_, and PbCl_2_.

**Table 2:** Energy parameters of PbCl_2_, PbCO_3_, PbSO_4_, Pb_3_(PO_4_)_2_, Pb_3_Fe_2_(PO_4_)_4_ and PbAl_2_O_4._

Name	Fermi level (eV)	Free energy (J/g atom)
PbCl_2_	1.6568	− 46.752487
PbCO_3_	1.9895	− 137.468327
PbSO_4_	2.6375	− 147.306324
Pb_3_(PO_4_)_2_	3.4376	− 177.837286
Pb_3_Fe_2_(PO_4_)_4_	3.1226	− 334.323287
PbAl_2_O_4_	2.4952	− 94.8346858

### Band structure and partial density of states

**Figure 2:**
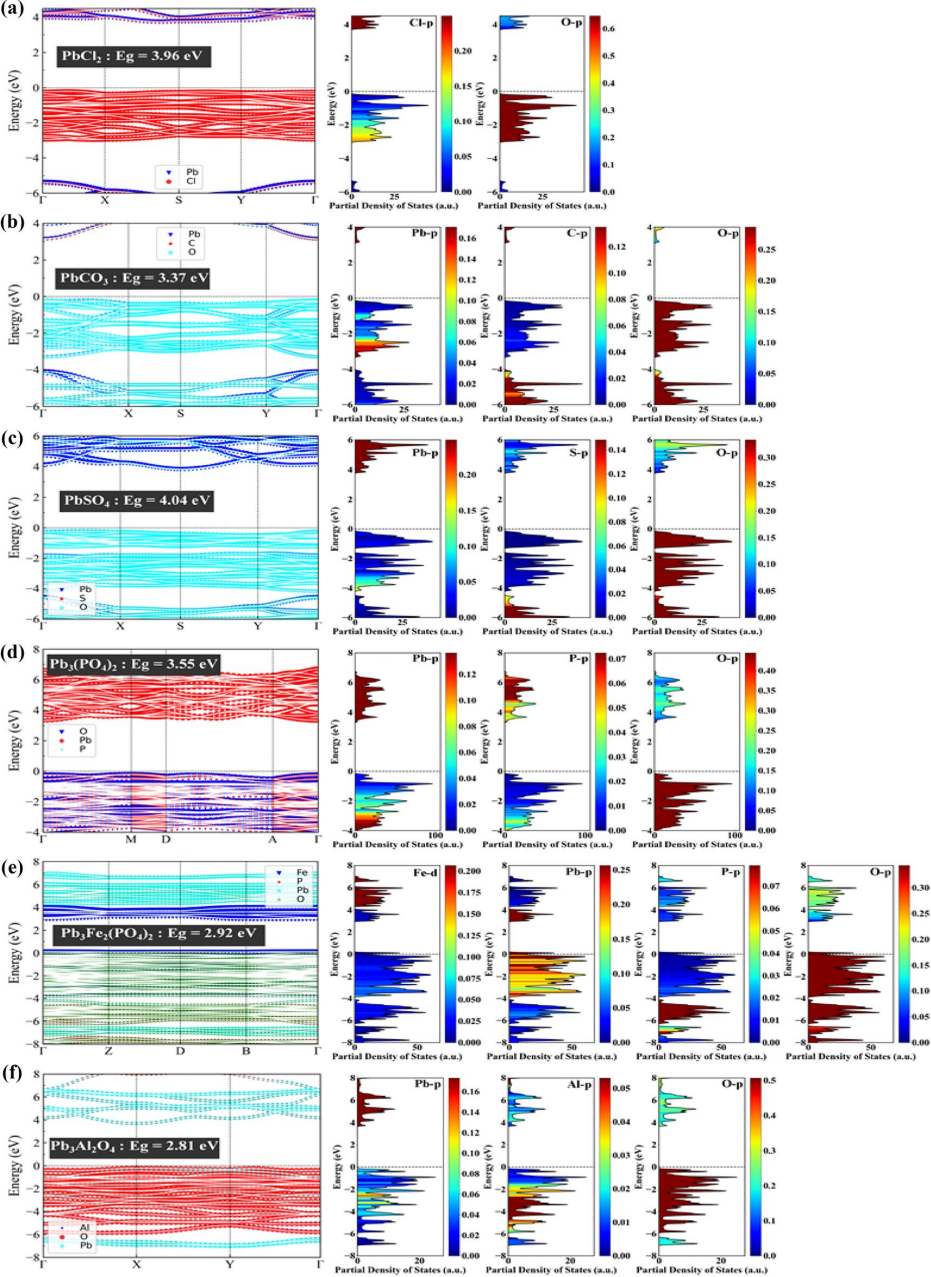
Band structure and state density distribution of PbCl_2_, PbCO_3_, PbSO_4_, Pb_3_(PO_4_)_2_, Pb_3_Fe_2_(PO_4_)_4_, and PbAl_2_O_4_(Note: 0 eV represents its corresponding Fermi level, in which Γ in abscissa is point Gamma, namely the center of Brillouin region).

By calculating the band structure of the above systems, deriving the band structure, and obtaining more detailed structural information about these systems, the electrical properties and crystal configuration of these systems could be deeply understood, and the functional groups of passivated lead pollution could be further determined. As shown in Fig. 2, the Fermi level (Ef) was taken as the energy zero. PbCl_2_, PbCO_3_, PbSO_4_, Pb_3_(PO_4_)_2_, Pb_3_Fe_2_(PO_4_)_4_, and PbAl_2_O_4_ had bandgaps of 3.96, 3.37, 4.04, 3.55, 2.92, and 2.81 eV, respectively. The valence band maximum of PbCl_2_, PbCO_3_, PbSO_4_, and Pb_3_(PO_4_)_2_ were all closer to their corresponding Fermi levels (the position of 0 eV in the figure). Thus were all p-type semiconductors. Therefore, these four systems are more likely to change their properties by attracting electrons. In addition, the overlap of the hybridized orbitals caused a high number of effective electrons and a high relative mass, resulting in a narrow bandgap for these four systems. The influence of the elements and orbitals in the system on the electronic structure could be seen from the partial density of states (PDOS) contribution diagram in Fig. 2. The conduction band of PbCl_2_ was dominated by the Cl-p orbital contribution and the valence band by the O-p orbital contribution, with the Cl-p and O-p orbitals overlapping at low energy levels. In the electronic structure of PbCO_3_, the conduction band was mainly contributed by the Pb-p and C-p orbitals, whereas the valence band was mostly contributed by the O-p orbital. Among them, the Pb-p and O-p orbitals overlapped at low energy levels, and the C-p and O-p orbitals overlapped at even lower energy levels. The conduction band of PbSO4 was mainly contributed by the Pb-p orbital, whereas the valence band was mainly contributed by the O-p orbital, with some contribution from S-p at the lower energy levels. In this system, S-p and O-p overlapped at low energy levels. The conduction band of Pb_3_(PO_4_)_2_ was mainly contributed by the Pb-p and P-p orbitals, and the Pb-p and O-p orbitals contributed to the valence band. Pb-p and P-p overlapped in the conduction band, whereas Pb-p and O-p overlapped at the lower energy levels of the valence band. These systems fluctuated greatly in the direction from the center of Brillouin to the center of the quadrilateral face. Further attention should be paid to the selection and production of passivation that changes their structural properties. In this paper, PbAl_2_O_4_ and Pb_3_Fe_2_(PO_4_)_4_ compounds containing lead have been considered. The purpose of this approach was to test whether metal cations such as Al^3+^ ions and Fe^3+^ ions can enhance the stability of lead compounds in soil. After metal cations were added, the energy gap width of the lead-containing system was greatly reduced, and the conductivity was significantly enhanced. In Fig. 2f, the Fermi level of PbAl_2_O_4_ was closer to the conduction band, and changing the properties could make it an n-type semiconductor, showing the properties of more metals. This finding is mainly due to the shift of the conduction band to the lower energy level caused by the hybridization of Pb-p, Al-p, and O-p orbitals. The Fermi energy level of the Pb_3_Fe_2_(PO_4_)_4_ system in Fig. 2e entered the valence band, so it is an N-type semiconductor, mainly because the hybridization of O-p and Pb-p orbitals shifted the valence band in the direction of higher energy levels. Moreover, the conduction band in the two combined crystals fluctuated greatly. In these two combined crystals, electron migration or transport was dominant, and losing electrons in the modification process was easy. The conductivity of Pb_3_Fe_2_(PO_4_)_4_ was significantly enhanced compared with that of Pb_3_(PO_4_)_2_, reflecting that the system is more stable and that adding Fe^3+^ could make the system containing lead more stable.

PDOS could reflect the degree of Pb^2+^ and anion binding inside the crystal. Thus, it could be used to infer the potential structural stability of these compounds, understand the proportion of Pb^2+^ and anion groups in the state density of the unit cell, and infer their contribution. Figure 2 is another visualization of the band structure, and the medium density distribution map of the entire energy interval had local spikes in the conduction band part and the valence band part. In the conduction and valence band regions, PbCO_3_, PbSO_4_, PbCl_2_, Pb_3_(PO_4_)_2_, PbAl_2_O_4_, and Pb_3_Fe_2_(PO_4_)_4_ were most affected by the p orbital, followed by the s orbital, and the d orbital was relatively small. In the region where the state density spiked, electrons were also dense, and the corresponding energy band was relatively narrow, in line with the observed energy band law. For more intuitive analysis and discussion of the contribution and influence of Pb^2+^, CO3^2−^, SO4^2−^, Cl^−^, PO4^3−^, and other anion groups on the whole system, the PDOS of each system and the state density of Pb^2+^ and each anion group are plotted in Fig. 2. The PDOS distribution diagram combined with the band structure diagram showed that the dispersion of PbSO_4_ was significantly greater than that of PbCO_3_, PbCl_2_, and Pb_3_(PO_4_)_2_, indicating that the interaction between Pb^2+^ and SO4^2−^ was stronger. In the conduction band, Pb^2+^ had a greater influence, and in the valence band, the anionic groups, such as CO_3_^2−^, SO_4_^2−^, and Cl^−^, occupied a larger proportion in the crystal. According to the calculation of the original data of state density, in the distribution of state density of PbCO_3_, PbSO_4_, PbCl_2_, and Pb_3_(PO_4_)_2_, CO_3_^2−^ was 63.32%, SO_4_^2−^ was 58.4%, Cl^−^ was 49.23%, and PO_4_^3−^ was 54.21%, respectively, indicating that anions dominated in these systems. This finding demonstrated that anion plays a leading role in the stability of the lead-containing system. However, in PbAl_2_O_4_ and Pb_3_Fe_2_(PO_4_)_4_, the dispersion decreased and the gold properties increased. The proportion of PbAl_2_O_4_ state density was Al^3+^ (5.79%), Pb^2+^ (14.57%), and O^2−^ (49.78%). O^2−^ was the main part of PbAl_2_O_4_, and the compound had high free energy, indicating that simple metal oxides are not stable enough. The medium density of Pb_3_Fe_2_(PO_4_)_4_ was 55.27% for PO_4_^3−^, 9.97% for Pb^2+^, and 13.35% for Fe^3+^. PO_4_^3−^ accounted for most of the system and played a dominant role in the valence zone. The above analysis of state density exhibited that the contribution of anionic groups SO_4_^2−^ and PO_4_^3−^ was significantly superior to other anionic groups in the lead-containing system. The introduction of two cationic lead-containing systems indicated that adding Fe^3+^ to the lead-containing system could make the system more stable. The stability of Pb^2+^ binding with common ions could be determined by calculating the free energy, energy band, and state density of the compound. SO_4_^2−^ and PO_4_^3−^ were speculated to be suitable functional groups to control Pb^2+^ pollution, and Fe^3+^ could increase the stability. However, a large number of them could also cause pollution. Therefore, substances that could release SO_4_^2−^ and PO_4_^3−^ easily were selected as suitable passivating agents.

The combination of PDOS and band structure diagram can more accurately reflect the crystal structure characteristics. PDOS can directly reflect the influence of different anion groups on the electron density of the whole compound system. The size of PDOS reflects the degree of binding between cations and anions in the system, and based on this, the stability of the compound can be predicted. In Fig. 2, PbCl_2_, PbCO_3_, PbSO_4_, Pb_3_(PO_4_)_2_, Pb_3_Fe_2_ (PO_4_). The electron density in these areas is greater than that in other locations in the crystal. The aggregation law was presented, and the narrow band in the corresponding location shown in the band structure mutually corroborated.

The figure shows that the dispersion of the valence band of the PbSO_4_ system was higher than that of PbCO_3_, PbCl_2_ and Pb_3_(PO_4_)_2_, suggesting that the binding force between lead ions and sulfate ionic groups was stronger. According to the original PDOS value obtained by calculation, the contribution rate of electron density in the six systems was the highest in the p orbital, followed by the s orbital, and the relative contribution rate of the d orbital was the lowest. In the four systems of PbCO_3_, PbSO_4_, PbCl_2_, and Pb_3_(PO_4_)_2_, CO_3_^2−^ accounted for 62.00%, SO_4_^2−^ accounted for 59.40%, and Cl^−^ accounted for 47.32% of the total energy of the corresponding system. Meanwhile, PO_4_^3−^ accounted for 53.99% of the total energy of the corresponding system. Excluding 10%-20% of the uncalculated electron energy in the total energy, the anionic group made a higher contribution to the whole system in these four systems. Therefore, the anionic group had a stronger stability certainty for the whole system. However, the dispersion of PbAl_2_O_4_ and Pb_3_Fe_2_(PO_4_)_4_ systems with other metal cations decreased significantly (the peak appeared more prominent), and the gold properties increased significantly. In the PbAl_2_O_4_ system, the PDOS energy accounted for 5.29% of the total energy of Al^3+^, 13.61% of the total energy of Pb^2+^, and 47.87% of the total energy of O^2−^. The anions accounted for the majority of the energy system of electrons, but their free energy was higher than that of other systems. This finding indicated that the oxides containing lead and other metal cations similar to PbAl_2_O_4_ had low stability. In the Pb_3_Fe_2_(PO_4_)_4_ system, the PDOS energy accounted for 55.18% of the total energy of PO_4_^3−^, 9.79% of the total energy of Pb^2+^, and 12.33% of the total energy of Fe^3+^. The phosphate anionic group accounted for most of the total energy of the electron, especially in the valence band. The distribution of PDOS above demonstrated that sulfate and phosphoric acid could be used as the preferred functional group for the passivation of lead pollution. When the passivation efficiency of sulfate and phosphoric acid is low, ferric ion could be considered to increase the stability of the system and achieve the purpose of passivation.

### Test application

The results of first-principle calculation showed that the preferred functional groups for lead pollution control are PO_4_^3−^ and SiO_3_^2−^, and the preferred functional groups for lead pollution control are SiO_3_^2−^ and PO_4_^3−^; SO_4_^2−^ could also be selected as treatment groups. When the selected process level is mature, the cation exchange of Na_2_HPO_4_·12H_2_O, Na_2_SO_4_, and Na_2_SiO_3_·9H_2_O is more likely to occur. Although the sample production process is mature, it could face the problems of counterfeit and high cost when used in large quantities. Therefore, the cheap industrial byproduct desulfurized gypsum (the main component is the same as natural gypsum, CaSO_4_·2H_2_O, content ≥ 93%) could also be used as passivating agent.

Test the calculation results through experiments to verify the effectiveness of the three passivating agents mentioned above in controlling lead pollution. Collect pollution-free soil from the Guanzhong area, air dry and grind it to 2 mm for later use. Prepare lead contaminated soil, dissolve 1500 mg/kg of PbCl_2_ (3 times the lead pollution control value) into water, and pour evenly into the test soil. Place the treated soil in a constant temperature and humidity incubator for aging treatment to shorten the experimental time. The initial mass fraction of Pb^2+^in the tested soil is 1347.2 mg/kg. Add Na_2_HPO_4_·12H_2_O, Na_2_SO_4_, and CaSO_4_·2H_2_O (93% purity) that need to control 2% soil quality to the self-made contaminated soil. Measure the mass fraction of available lead in the soil after 3, 5, and 15 days of treatment. The measurement results are shown in Table 3.

**Table 3:** Active mass fraction of lead after passivating agent.

Time/d	Anhydrous sodium sulfate	Sodium silicate	Desulfurization gypsum
0	1347.2	1347.2	1347.2
3	1182.48	1203.45	1269.64
5	1053.24	1088.36	1158.05
15	946.63	1013.51	1107.55

Table 3 shows that the three passivating agents had significant treatment effect on Pb^2+^ in polluted soil, and they could reduce the mass fraction of available lead by 28.56%–39.23% in 15 days. Among them, anhydrous sodium sulfate had the best effect, which verified that SO_4_^2−^ and SiO_3_^2−^ could be used as effective functional groups to control the passivating agents of lead pollution.

## Discussion

The control of soil Pb pollution has always been a hot topic in scientific research, and soil health is related to the lifeblood of human development^28^. Existing studies mostly focused on the remediation of contaminated soil through experimental means^29,30^. These methods take into account the characteristics of biological carbon with high activity and large specific surface area, which could absorb heavy metal elements in soil. However, research on the characteristics of lead compounds is insufficient. Rizwan et al. immobilized lead in polluted soil by using organic or inorganic amendments^31^. Belviso et al. used waste slag to remediate polluted soil^32^. This similar method tried to solve the soil problem by reusing the existing waste materials, but the internal mechanism of the effect has not been explored in depth^33,34^. Some scholars attempted to introduce first principles in physics to solve the problem of soil heavy-metal pollution^35,36^. Balan et al. analyzed the release kinetics of lead from soil, and Zhang et al. analyzed the adsorption of kaolinite on Mg, Sb, Cu, As, Al, and other heavy-metal elements^37,38^. However, no specific description of the specific role of compounds in kaolinite and perovskite could be found.

Qin et al. defined the ecological risk threshold of lead in soil in China^39^. Jensen et al. studied the existence of lead in polluted soil but did not conduct an in-depth analysis of lead compounds^40^. In the present paper, by analyzing the geometry, electronic structure, density of states, charge transfer, and energy conversion of different lead compound crystals, the internal mechanism of different lead compounds in the passivation process was clarified, and the passivation ability of different lead compounds was explored. The calculation results were verified by auxiliary experiments, and they are consistent with the research results of Kong et al., In the present study, the addition of Fe ions could enhance the passivation effect of sulfate and phosphate. This conclusion confirmed the experimental results of Teng et al. on Fe ion-modified rice husks to fix lead compounds^41^.

Using first-principle to study the stability of five different lead compounds is a new attempt to address soil heavy-metal pollution, and it provides interesting insights into the enhanced understanding of the stability and transformation of heavy metal elements in nature. By calculating the crystal structure of lead compounds and analyzing the reaction between lead-contaminated soil and different ions, the defects of experimental chemistry are made up, and the internal mechanism of passivation of lead-contaminated soil and the treatment of soil heavy metal pollution are provided.

## Conclusion

The order of Gibbs free energy of the six lead-containing systems from small to large is Pb_3_Fe_2_(PO_4_)_4_, Pb_3_(PO_4_)_2_, PbSO_4_, PbCO_3_, PbAl_2_O_4_, and PbCl_2_. The relative stability of these systems is inversely correlated with the corresponding Gibbs free energy order. The structural stability of this series of lead compounds in descending order is as follows: Pb_3_Fe_2_(PO_4_)_4_, Pb_3_(PO_4_)_2_, PbSO_4_, PbCO_3_, PbAl_2_O_4_, and PbCl_2_.

A comprehensive analysis of the band structure and PDOS showed that lead carbonate, lead sulfate, lead chloride, and lead phosphate are all p-type semiconductors. The crystal structure of this series of systems was not filled with electrons, and obtaining electrons to fill the crystal holes is easy when the properties change. After metal cations were added, the PbAl_2_O_4_ system was identified to be an n-type semiconductor, whereas the Pb_3_Fe_2_(PO_4_)_4_ system was found to be a p-type semiconductor. These two lead systems containing common metal cations showed obvious metal properties. The electrons in the lattice are more active and could migrate and conduct electricity in the lattice, which could easily lose electrons in the crystal when the properties change.

Based on the analysis of ionic groups, such as CO_3_^2−^, SO_4_^2−^, Cl^−^, PO_4_^3−^, Al^3+^, and Fe^3+^, sulfate and phosphoric acid could be used as the preferred functional groups for lead pollution passivation. When the efficiency of sulfate and phosphoric acid passivation is low, adding ferric ions could be considered to increase the stability of the system to achieve the purpose of passivation.

## Data Availability

The datasets used and/or analysed during the current study available from the corresponding author on reasonable request.
